# *catena*-Poly[[bis­(1*H*-imidazole-κ*N^3^*)copper(II)]-μ-benzene-1,4-dicarboxyl­ato-κ^2^ *O* ^1^:*O* ^4^]

**DOI:** 10.1107/S1600536811024822

**Published:** 2011-07-02

**Authors:** Qian Xu

**Affiliations:** a Ordered Matter Science Research Center, College of Chemistry and Chemical Engineering, Southeast University, Nanjing 211189, People’s Republic of China

## Abstract

In the title compound, [Cu(C_8_H_4_O_4_)(C_3_H_4_N_2_)_2_]_*n*_, the Cu^II^ atom is four-coordinated by two carboxyl­ate O atoms from two different terephthalate ligands and two N atoms from two imidazole ligands in a slightly distorted square-planar coordination environment. Each terephthalate ligand acts as a bis-monodentate ligand that binds two Cu^II^ atoms, thus forming two unique chains extending parallel to [110]. The imidazole ligands are attached on both sides of the chains.

## Related literature

For general background to ferroelectric metal-organic compounds with framework structures, see: Fu *et al.* (2009 ▶); Ye *et al.* (2006 ▶); Zhang *et al.* (2008 ▶, 2010 ▶).
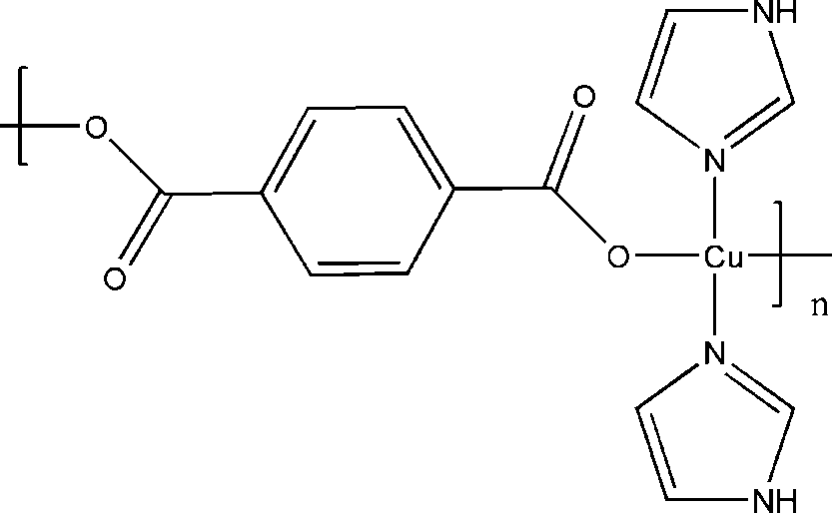


## Experimental

### 

#### Crystal data

[Cu(C_8_H_4_O_4_)(C_3_H_4_N_2_)_2_]
*M* *_r_* = 363.82
Monoclinic, 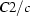

*a* = 21.435 (4) Å
*b* = 5.2740 (11) Å
*c* = 14.164 (3) Å
β = 116.65 (3)°
*V* = 1431.1 (5) Å^3^
*Z* = 4
Mo *K*α radiation
μ = 1.55 mm^−1^
*T* = 293 K
0.30 × 0.25 × 0.20 mm

#### Data collection

Rigaku SCXmini diffractometer
Absorption correction: multi-scan (*CrystalClear*; Rigaku, 2005 ▶) *T*_min_ = 0.634, *T*_max_ = 0.733
6976 measured reflections
1641 independent reflections
1440 reflections with *I* > 2σ(*I*)
*R*_int_ = 0.048

#### Refinement

*R*[*F* ^2^ > 2σ(*F* ^2^)] = 0.033
*wR*(*F* ^2^) = 0.085
*S* = 1.08
1641 reflections
110 parameters
H atoms treated by a mixture of independent and constrained refinement
Δρ_max_ = 0.26 e Å^−3^
Δρ_min_ = −0.32 e Å^−3^

### 

Data collection: *CrystalClear* (Rigaku, 2005 ▶); cell refinement: *CrystalClear*; data reduction: *CrystalClear*; program(s) used to solve structure: *SHELXS97* (Sheldrick, 2008 ▶); program(s) used to refine structure: *SHELXL97* (Sheldrick, 2008 ▶); molecular graphics: *SHELXTL* (Sheldrick, 2008 ▶); software used to prepare material for publication: *SHELXL97*.

## Supplementary Material

Crystal structure: contains datablock(s) I, global. DOI: 10.1107/S1600536811024822/jh2303sup1.cif

Structure factors: contains datablock(s) I. DOI: 10.1107/S1600536811024822/jh2303Isup2.hkl

Additional supplementary materials:  crystallographic information; 3D view; checkCIF report

## Figures and Tables

**Table 1:** Hydrogen-bond geometry (Å, °)

*D*—H⋯*A*	*D*—H	H⋯*A*	*D*⋯*A*	*D*—H⋯*A*
N2—H2*A*⋯O1^i^	0.78 (4)	2.11 (4)	2.851 (3)	157 (4)

Symmetry code: (i) 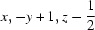.

**Fig. 1.**
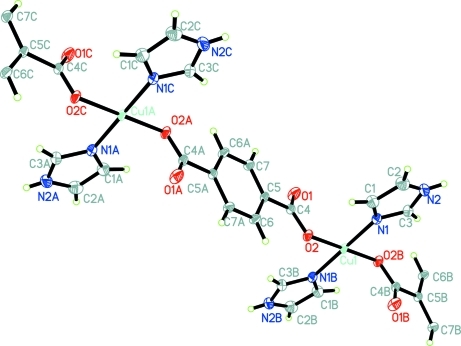
The molecular structure of the title compound, showing the atomic numbering scheme. Displacement ellipsoids are drawn at the 30% probability level. Symmetry codes for A: 1 - x, 2 - y, 2 - z. Symmetry codes for B: 1.5 - x, 1.5 - y, 2 - z. Symmetry codes for C: 0.5 - x, 2.5 - y, 2 - z.

**Fig. 2.**
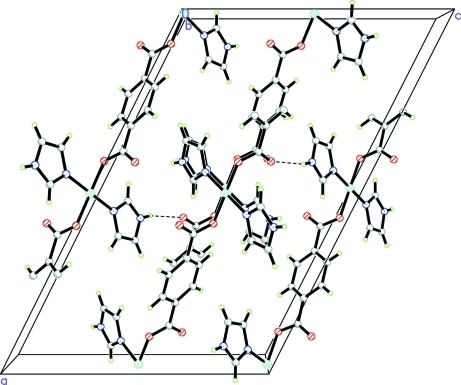
A view of the packing of the title compound, stacking along the b axis. Dashed lines indicate hydrogen bonds.

## supplementary crystallographic information

### Comment

Dielectric constant measurements of compounds as a function of temperature is the basic method to find potential ferroelectric phase change materials (Fu *et al.*, 2009; Ye *et al.*, 2006; Zhang *et al.*, 2008; Zhang *et al.*, 2010). Unfortunately, the study carried out on the title compound indicated that the permittivity is temperature-independent, suggesting that there may be no dielectric disuniformity between 80 K to 350 K (m.p. 393–381 K). In this report the crystal structure of the title compound is reported.

An *ORTEP* diagram showing the structure of the title compound with the symmetry related fragments and atom-numbering scheme is shown in Fig. 1. The Cu(II) atom,with an inversion center, adopts a distorted octahedral geometry comprising two nitrogen atoms of two pyrazole ligands [Cu1–N1 = 1.9859 (19) Å] and two oxygen atoms of two different tp ligands [Cu1–O1 = 1.9408 (14) Å]. The N2O2 moiety defines the equatorial plane of the octahedron. In the equatorial plane, the angles between the *cis*-positioned atoms are close to 90° and those of the transpositioned atoms are identical to 180° since the Cu atom resides at the inversion center.

### Experimental

An aqueous solution of imidazole (1.64 g, 20 mmol) and terephthalic acid (10 mmol) was treated with CuCl_2_(1.35 g, 10 mmol). After the mixture was churned for a few minutes, slow evaporation of the resulting solution yielded blue crystals after a few days.

### Refinement

All H atoms were placed in geometrically idealized positions and constrained to ride on their parent atoms except H2A with C—H = 0.93 Å, with *U*_iso_(H) = 1.2 *U*_iso_(C).

### Crystal data

**Table:**

[Cu(C_8_H_4_O_4_)(C_3_H_4_N_2_)_2_]	*Z* = 4
*M**_r_* = 363.82	*F*(000) = 740
Monoclinic, *C*2/*c*	*D*_x_ = 1.689 Mg m^−^^3^
Hall symbol: -C 2yc	Mo *K*α radiation, λ = 0.71073 Å
*a* = 21.435 (4) Å	θ = 2.8–27.5°
*b* = 5.2740 (11) Å	µ = 1.55 mm^−^^1^
*c* = 14.164 (3) Å	*T* = 293 K
β = 116.65 (3)°	Block, blue
*V* = 1431.1 (5) Å^3^	0.30 × 0.25 × 0.20 mm

### Data collection

**Table:**

Rigaku SCXmini diffractometer	1641 independent reflections
Radiation source: fine-focus sealed tube	1440 reflections with *I* > 2σ(*I*)
graphite	*R*_int_ = 0.048
CCD_Profile_fitting scans	θ_max_ = 27.5°, θ_min_ = 4.0°
Absorption correction: multi-scan (*CrystalClear*; Rigaku, 2005)	*h* = −27→27
*T*_min_ = 0.634, *T*_max_ = 0.733	*k* = −6→6
6976 measured reflections	*l* = −18→18

### Refinement

**Table:**

Refinement on *F*^2^	Secondary atom site location: difference Fourier map
Least-squares matrix: full	Hydrogen site location: inferred from neighbouring sites
*R*[*F*^2^ > 2σ(*F*^2^)] = 0.033	H atoms treated by a mixture of independent and constrained refinement
*wR*(*F*^2^) = 0.085	*w* = 1/[σ^2^(*F*_o_^2^) + (0.040*P*)^2^ + 0.9748*P*] where *P* = (*F*_o_^2^ + 2*F*_c_^2^)/3
*S* = 1.08	(Δ/σ)_max_ < 0.001
1641 reflections	Δρ_max_ = 0.26 e Å^−^^3^
110 parameters	Δρ_min_ = −0.32 e Å^−^^3^
0 restraints	Extinction correction: *SHELXL97* (Sheldrick, 2008)
Primary atom site location: structure-invariant direct methods	Extinction coefficient: 0

### Special details

**Table:**

Geometry. All e.s.d.'s (except the e.s.d. in the dihedral angle between two l.s. planes) are estimated using the full covariance matrix. The cell e.s.d.'s are taken into account individually in the estimation of e.s.d.'s in distances, angles and torsion angles; correlations between e.s.d.'s in cell parameters are only used when they are defined by crystal symmetry. An approximate (isotropic) treatment of cell e.s.d.'s is used for estimating e.s.d.'s involving l.s. planes.
Refinement. Refinement of *F*^2^ against ALL reflections. The weighted *R*-factor *wR* and goodness of fit *S* are based on *F*^2^, conventional *R*-factors *R* are based on *F*, with *F* set to zero for negative *F*^2^. The threshold expression of *F*^2^ > σ(*F*^2^) is used only for calculating *R*-factors(gt) *etc*. and is not relevant to the choice of reflections for refinement. *R*-factors based on *F*^2^ are statistically about twice as large as those based on *F*, and *R*- factors based on ALL data will be even larger.

### Fractional atomic coordinates and isotropic or equivalent isotropic displacement parameters (Å^2^)

**Table:**

	*x*	*y*	*z*	*U*_iso_*/*U*_eq_	
C1	0.37485 (12)	0.7554 (5)	0.82142 (19)	0.0385 (5)	
H1	0.3432	0.8726	0.8240	0.046*	
C2	0.35883 (15)	0.5594 (5)	0.7531 (2)	0.0460 (6)	
H2	0.3148	0.5180	0.7002	0.055*	
C3	0.47038 (14)	0.5553 (5)	0.8563 (2)	0.0387 (6)	
H3	0.5170	0.5067	0.8866	0.046*	
C4	0.38692 (10)	1.0497 (4)	1.04391 (18)	0.0269 (5)	
C5	0.31559 (10)	1.1531 (4)	1.02048 (16)	0.0249 (4)	
C6	0.29077 (11)	1.3731 (4)	0.96075 (18)	0.0303 (5)	
H6	0.3179	1.4565	0.9344	0.036*	
C7	0.27444 (12)	1.0321 (4)	1.05946 (19)	0.0301 (5)	
H7	0.2908	0.8856	1.0997	0.036*	
N1	0.44579 (9)	0.7532 (3)	0.88681 (14)	0.0303 (4)	
N2	0.41890 (14)	0.4355 (5)	0.77617 (19)	0.0447 (6)	
O1	0.41430 (8)	0.8830 (3)	1.11055 (14)	0.0426 (4)	
O2	0.41356 (7)	1.1465 (3)	0.98747 (12)	0.0306 (3)	
Cu1	0.5000	1.0000	1.0000	0.02373 (14)	
H2A	0.4238 (19)	0.322 (7)	0.744 (3)	0.074 (11)*	

### Atomic displacement parameters (Å^2^)

**Table:**

	*U*^11^	*U*^22^	*U*^33^	*U*^12^	*U*^13^	*U*^23^
C1	0.0315 (12)	0.0408 (13)	0.0383 (13)	0.0008 (10)	0.0112 (10)	−0.0025 (11)
C2	0.0446 (16)	0.0497 (15)	0.0364 (14)	−0.0083 (12)	0.0118 (12)	−0.0049 (11)
C3	0.0433 (14)	0.0348 (12)	0.0468 (15)	0.0008 (10)	0.0281 (12)	−0.0052 (10)
C4	0.0204 (10)	0.0273 (10)	0.0356 (12)	0.0022 (8)	0.0149 (9)	−0.0036 (8)
C5	0.0185 (9)	0.0276 (10)	0.0313 (11)	0.0029 (7)	0.0136 (8)	−0.0019 (8)
C6	0.0234 (10)	0.0324 (11)	0.0412 (12)	0.0028 (9)	0.0200 (9)	0.0061 (10)
C7	0.0252 (11)	0.0280 (11)	0.0411 (13)	0.0065 (8)	0.0184 (10)	0.0076 (9)
N1	0.0278 (9)	0.0327 (9)	0.0348 (10)	0.0012 (7)	0.0179 (8)	−0.0024 (8)
N2	0.0651 (16)	0.0371 (11)	0.0443 (13)	−0.0095 (11)	0.0354 (12)	−0.0117 (10)
O1	0.0308 (8)	0.0477 (10)	0.0555 (11)	0.0185 (8)	0.0248 (8)	0.0186 (9)
O2	0.0226 (7)	0.0345 (8)	0.0408 (9)	0.0066 (6)	0.0197 (7)	0.0009 (7)
Cu1	0.0187 (2)	0.0272 (2)	0.0299 (2)	0.00423 (13)	0.01502 (16)	0.00013 (14)

### Geometric parameters (Å, °)

**Table:**

C1—C2	1.351 (4)	C5—C7	1.388 (3)
C1—N1	1.381 (3)	C5—C6	1.393 (3)
C1—H1	0.9300	C6—C7^i^	1.388 (3)
C2—N2	1.347 (4)	C6—H6	0.9300
C2—H2	0.9300	C7—C6^i^	1.388 (3)
C3—N1	1.326 (3)	C7—H7	0.9300
C3—N2	1.335 (4)	N1—Cu1	1.9857 (19)
C3—H3	0.9300	N2—H2A	0.78 (4)
C4—O1	1.229 (3)	O2—Cu1	1.9408 (14)
C4—O2	1.279 (3)	Cu1—O2^ii^	1.9408 (14)
C4—C5	1.513 (3)	Cu1—N1^ii^	1.9857 (19)
			
C2—C1—N1	108.9 (2)	C5—C6—H6	120.0
C2—C1—H1	125.5	C6^i^—C7—C5	120.9 (2)
N1—C1—H1	125.5	C6^i^—C7—H7	119.6
N2—C2—C1	106.8 (2)	C5—C7—H7	119.6
N2—C2—H2	126.6	C3—N1—C1	105.5 (2)
C1—C2—H2	126.6	C3—N1—Cu1	127.21 (17)
N1—C3—N2	110.6 (2)	C1—N1—Cu1	127.28 (15)
N1—C3—H3	124.7	C3—N2—C2	108.2 (2)
N2—C3—H3	124.7	C3—N2—H2A	125 (3)
O1—C4—O2	124.91 (19)	C2—N2—H2A	126 (3)
O1—C4—C5	120.90 (19)	C4—O2—Cu1	117.36 (13)
O2—C4—C5	114.17 (18)	O2^ii^—Cu1—O2	180.000 (1)
C7—C5—C6	119.12 (18)	O2^ii^—Cu1—N1	90.10 (7)
C7—C5—C4	120.45 (19)	O2—Cu1—N1	89.90 (7)
C6—C5—C4	120.42 (18)	O2^ii^—Cu1—N1^ii^	89.90 (7)
C7^i^—C6—C5	120.00 (19)	O2—Cu1—N1^ii^	90.10 (7)
C7^i^—C6—H6	120.0	N1—Cu1—N1^ii^	180.0

Symmetry codes: (i) −*x*+1/2, −*y*+5/2, −*z*+2; (ii) −*x*+1, −*y*+2, −*z*+2.

### Hydrogen-bond geometry (Å, °)

**Table:**

*D*—H···*A*	*D*—H	H···*A*	*D*···*A*	*D*—H···*A*
N2—H2A···O1^iii^	0.78 (4)	2.11 (4)	2.851 (3)	157 (4)

Symmetry codes: (iii) *x*, −*y*+1, *z*−1/2.
